# Pulmonary vein isolation durability after very high-power short-duration ablation utilizing a very-close protocol – The FAST AND FURIOUS redo study

**DOI:** 10.1016/j.ijcha.2023.101325

**Published:** 2023-12-20

**Authors:** Christian-H. Heeger, Behnam Subin, Charlotte Eitel, Sorin Ștefan Popescu, Huong-Lan Phan, Roman Mamaev, Lorenzo Bartoli, Niels Große, Samuel Reincke, Anna Traub, Delgado Lopez, Bettina Kirstein, Sascha Hatahet, Karl-Heinz Kuck, Julia Vogler, Roland R. Tilz

**Affiliations:** a University Heart Center Lübeck, Department of Rhythmology, University Hospital Schleswig-Holstein, Germany; b German Center for Cardiovascular Research (DZHK), Partner Site Hamburg/Kiel/Lübeck, Lübeck, Germany

**Keywords:** Atrial fibrillation, high-power short duration, pulmonary vein Isolation, Radiofrequency, Durability

## Abstract

**Background:**

Very high-power short-duration (vHP-SD) radiofrequency (RF) ablation of atrial fibrillation (AF) treatment by pulmonary vein isolation (PVI) aims for safer, more effective and faster procedures. Although acute efficacy and safety for PVI was recently shown data on chronic PVI durability is limited. Here chronic PVI durability was evaluated during repeat electrophysiological procedures in patients after initial vHP-SD and conventional RF based PVI.

**Methods:**

A total of 25 consecutive patients with repeat left atrial procedures after initial vHP-SD based PVI were included in this study. Twenty-five patients with previous conventional RF based PVI and repeat left atrial procedures served as control (control group).

**Results:**

For index procedures the median RF time was 328 (277, 392) seconds (vHP-SD) and 1470 (1310, 1742) seconds (control); p < 0.001, the median procedure time was 55 (53, 68) minutes (vHP-SD) and 110 (94, 119) (control); p < 0.001). First pass isolation rate was 84 % (vHP-SD) and 88 % (control, p = 0.888). No differences for severe adverse events (vHP-SD: 1/25, 4 % vs. control: 0/25, 0 %; p = 0.676 were detected.

Chronic durability of all PVs was assessed in vHP-SD: 16/25 (64 %) and control: 8/25 (32 %) patients (p = 0.023) and vHP-SD: 81 % and control: 62 % of PVs were found to be isolated (p = 0.003). For right PVs vHP-SD: 84 % vs. control: 60 % of PVs (p < 0.001) and for left PVs vHP-SD: 78 % vs. control: 64 % (p = 0.123) were found to be isolated.

**Conclusions:**

PVI solely utilizing vHP-SD via a very close-protocol provides fast, safe and effective acute PVI. High rates of chronically isolated pulmonary veins have been detected.

## 1 Introduction

Catheter ablation for treatment of paroxysmal (PAF) and persistent atrial fibrillation (PersAF) by pulmonary vein isolation (PVI) has proven high procedural and long-term success rates.[1] Recently single-shot ablation systems showed similar findings with decreased procedure time and safety compared to radiofrequency (RF) based 3D mapping and point-by-point PVI.[2] Nevertheless single-shot systems are mainly designed for PVI only and lacks the adaptability to different PV anatomies as well as treatment options for different cardiac arrythmias. Single-tip ablation catheters received several improvements by implementing contact force (CF) sensing and ablation index (AI) guided RF ablation.[3], [4] Recently very high-power short-duration (vHP-SD) catheter ablation with a maximum of 90Watts for 4 s have been evaluated and safety, efficacy and efficiency have been shown.[5], [6] The QDOT Micro ablation catheter (Biosense Webster, Inc. Diamond Bar, CA, USA) has been developed for vHP-SD ablation controlled by real-time assessment of catheter-to-tissue temperature and therefore allows for temperature-controlled ablation.[7], [8] Since the lesion formation of vHP-SD ablation creates wider but shallower lesions the close-protocol was adapted to an individualized and tighter “very close-protocol” using vHP-SD only.[9] Data on chronic PVI durability was recently assessed by cardiac MRI in patients previously treated by vHP-SD.[10] The findings are promising and are in line with findings recently assessed in the FAST and FURIOUS PVI study.[9] Nevertheless, patients number were relatively low. The FAST and FURIOUS Redo study aims to assess data of PVI durability after initial vHP-SD very close protocol based PVI in comparison to conventional RF based PVI.

## 2 Methods

### 2.1 Inclusion and exclusion criteria

All patients with repeat left atrial procedures after initial vHP-SD based PVI were included in this study (vHP-SD group). A total of 25 consecutive previous patients treated with conventional CF-sensing AI guided PVI and repeat left atrial procedures served as control (control group). The patients were prospectively and consecutively enrolled. Exclusion criteria were prior left atrial (LA) ablation attempts, LA diameter > 60 mm, severe valvular heart disease or contraindications to post-interventional oral anticoagulation. Transesophageal echocardiography was performed in all patients prior to PVI to rule out intracardiac thrombi and to assess the LA diameter. No further pre-procedural imaging was performed. In patients on vitamin K antagonists the procedure was performed under therapeutic INR values of 2–3. In patients on new oral anticoagulants the morning dose on the day of the procedure was omitted. All patients gave written informed consent and all patient information was anonymized. The study was approved by the local ethics board (Lübeck ablation registry ethical review board number: WF-028/15) and performed in accordance to the ethical standards laid down in the 1964 Declaration of Helsinki and its later amendments.

### 2.2 Intraprocedural management: Index procedure

The detailed intraprocedural management for vHP-SD as well as AI guided PVI has been described in previous studies from our group.[9], [11] In brief, the procedure was performed under deep sedation using midazolam, fentanyl and propofol. Three ultrasound guided right femoral vein punctures were performed and three 8F short sheaths were inserted. Prior to transseptal puncture one diagnostic catheter was introduced and positioned inside the coronary sinus. Double transseptal puncture (TSP) was performed under fluoroscopic guidance using a modified Brockenbrough technique with 8.5F transseptal sheaths and puncture needle (SL1 sheath and BRK-1 TSP needle, St. Jude Medical, Inc., St. Paul, MN, USA). Pulmonary vein (PV) angiography was performed to identify the PV ostia. Both sheaths were continuously flushed with heparinized saline (10 ml/h). After TSP heparin boluses were administered targeting an activated clotting time of > 300 s.

### 2.3 Ablation procedure

Three-dimensional (3D) electroanatomic LA reconstruction (CARTO 3 V7, Biosense Webster) was performed via fast anatomical mapping with a multi-electrode mapping catheter (Pentaray, Octaray or Lasso Nav, Biosense Webster). For the LA voltage map, the bipolar voltage reference interval was set between 0.05 and 0.5 mV. After PV angiography the ipsilateral PVs were tagged according to 3D mapping and PV angiography. During PVI a multi-electrode mapping catheter was positioned inside the ipsilateral PVs.

**Fig. 1:**
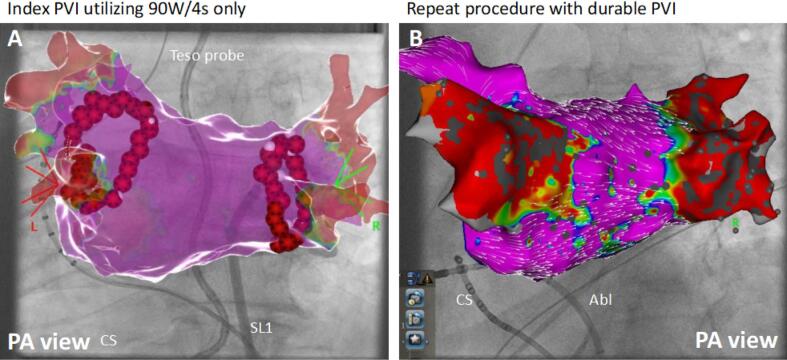
Final lesions set and repeat procedure after vHP-SD based PVI A: Three-dimensional electroanatomical reconstruction (CARTO 3, UNIVIEW module, Biosense Webster) of the left atrium in posterior anterior (left) view. Please note the two circles of very-high power short duration applications by 90 W/4 s (QMODE + mode, red–black tags) encircling the right and left pulmonary veins. A very close protocol was utilized: At the posterior area an inter-lesion distance of 5–6 mm was targeted and at the anterior area an inter-lesion distance of 3–4 mm was targeted. B: Three-dimensional electroanatomical reconstruction of the left atrium in posterior anterior view of the same patient (A) after 12 months. Please note the durable isolated right and left pulmonary veins. (For interpretation of the references to colour in this figure legend, the reader is referred to the web version of this article.)

In the vHP-SD group the QDOT Micro catheter was utilized as recently described.[9] For all applications vHP-SD ablation utilizing the QMODE + ablation mode was performed. The target temperature of the temperature-controlled ablation was 60 °C based on the hottest surface thermocouple.[7] A switch to conventional QMODE was always possible by changing the ablation mode. For all cases a very close protocol was utilized aiming to perform vHP-SD only: For anterior lesions an inter-lesion distance of 3–4 mm and for posterior lesions an inter-lesion distance of 5–6 mm was predefined. The target CF range was 10–25 g. The final lesion set after vHP-SD based PVI is shown in Fig. 1. An S-shaped temperature probe (CIRCA S-CATH, Circa Scientific, Englewood, CO, USA) was utilized to monitor the esophageal temperature (Teso) in all cases of the vHP-SD group. The intraluminal Teso cut-off was set at 38.5 °C. During the procedures special attention was drawn for audible pops and all catheters were checked for charring after removal.

In the control group conventional CF-sensing AI guided ablation was used. Ablation was performed with a Thermocool Smart-touch surround flow catheter (Biosense Webster) in a power-controlled mode. Energy application was limited to 40 W. Target range for CF was 10–40 g. Target AIs were 550, 450 and 380 for the anterior, roof and posterior segments of the LA, respectively.[11] The inter-lesion distance was set to 5–6 mm.

In case of previously known or periprocedural typical atrial flutter, cavotricuspid isthmus ablation was performed in both groups.

### 2.4 Postprocedural care

A figure-of-eight suture and a pressure bandage were used to prevent femoral bleeding. The pressure bandage was removed after 4 h and the figure-of-eight suture on the next day. Following ablation, all patients underwent transthoracic echocardiography immediately post procedure, after 1–2 h and at day 1 after the procedure to rule out a pericardial effusion. New oral anticoagulants were re-initiated 6 h post ablation. Anticoagulation was continued for at least 3 months and continued thereafter based on the patients CHA_2_DS_2_-VASc score. Previously ineffective antiarrhythmic drugs or a new antiarrhythmic drug were prescribed and continued for 3 months post ablation. All patients were treated with proton-pump inhibitors for 6 weeks. Following a 3-months blanking period, patients completed outpatient clinic visits, including ECG and 72 h-Holter ECG at 3, 6 and 12 months. In addition, regular telephone interviews were performed. Additional outpatient clinic visits were immediately initiated in cases of symptoms suggestive of arrhythmia recurrence. For all patients with AF / AT recurrence outside the blanking period a repeat electrophysiological procedure was suggested. All patients of the study group agreed to this proceeding.

### 2.5 Repeat procedures

Patients with AF or atrial tachycardia (AT) recurrence during the follow-up and suitable for a repeat-PVI as well as patients planed for LAA closure were scheduled for a second ablation procedure. The techniques for mapping and RF-based PVI have been previously described.[12] The procedures were performed as per institutional standards. Excluding the patients planned for LAA closure an LA electroanatomic reconstruction was performed using a multipolar mapping catheter and a 3D mapping system (Carto or EnsiteX). In the patients planned for LAA closure a multipolar mapping catheter was utilized for assessed of PVI durability. Each individual PV was evaluated for electrical reconnection using the mapping catheter recordings. For the LA voltage map, the bipolar voltage reference interval was set between 0.05 and 0.5 mV. After PV angiography the ipsilateral PVs were tagged according to 3D mapping and PV angiography. During PVI a multi-electrode mapping catheter was positioned inside the ipsilateral PVs. When non-isolated PVs were identified, a RF-based, point by point PVI was performed as per institutional standard.[12] For treatment of AT high density mapping utilizing a 3D-mapping system and a multipolar mapping catheter was conducted to identify the AT mechanism followed by deployment of standardized ablation lines as previously described.[11].

### 2.6 End points

The primary endpoint was defined as chronic PVI durability in all detected PVs. The secondary end points were: acute procedural success defined as the ability to confirm electrical isolation with a multipolar mapping catheter, procedural parameters (e.g. procedure time, LA dwelling time, fluoroscopy time), number and duration of RF applications, number of first pass isolations as well as periprocedural complications. Periprocedural complications were defined according to latest guidelines. Only adverse events adjudicated as possible, probable, or definitely related to the ablation procedure were mentioned as safety events. An adverse event was considered serious if it resulted in permanent injury or death, required an intervention for treatment, or required hospitalization for more than 24 h. All other safety events were defined as minor complications.

### 2.7 Statistical analysis

Continuous variables are presented as median with interquartile range (first quartile [Q1], third quartile [Q3]); they were compared using the Wilcoxon-Mann Whitney test. Categorical variables are presented as absolute and relative frequencies; they were compared using the chi-square test or Fisher’s exact test (in case of small expected cell frequencies). All p-values are two-sided and a p-value < 0.05 was considered significant. Recurrence-free survival was estimated with the Kaplan-Meier method. All calculations were performed with the statistical analysis software SAS (SAS Institute Inc., version 9.3, Cary, NC, USA).

## 3 Results

### 3.1 Patient characteristics

A total of 25 consecutive patients with previous vHP-SD based PVI utilizing the QMODE + ablation mode and a repeat left atrial procedure were included in this study (vHP-SD group). The data was compared to 25 consecutive previous patients with PVI by conventional CF-sensing AI guided ablation and a repeat left atrial procedure (control group). Patient baseline characteristics are shown in Table 1. No demographic differences were detected between the groups.

**Table 1:** Baseline patient characteristics.

**Variable**	**vHP-SD**	**Control**	**P**
Patients	25	25	
Age, years	71 (65, 77)	69 (64, 75)	0.415
LA volume, ml/m^2^*	30 (27, 35)	32 (29, 37)	0.655
Duration of AF, months	8 (3, 12)	9 (4, 14)	0.754
Female gender	11 (44)	12 (48)	0.777
Paroxysmal AF	12 (48)	14 (66)	0.571
Congestive heart failure	5 (20)	3 (12)	0.440
Arterial hypertension	14 (66)	11 (44)	0.396
Diabetes mellitus type 2	3 (12)	1 (4)	0.297
Coronary artery disease	2 (8)	1 (4)	0.552
Previous TIA / Stroke	1 (4)	0 (0)	0.676
CHA_2_DS_2_-VASc score			0.657
0	2	2	
1	6	4	
2	12	14	
3	4	3	
≥4	1	2	
HASBLED score			
0	1	2	
1	5	6	
2	14	13	
3	4	3	
≥4	1	1	

Values are counts, n (%) or mean (±SD).

AF = atrial fibrillation, LA = left atrium, TIA = transient ischaemic attack.

* per body surface area.

### 3.2 Procedural characteristics

Procedural data of the index procedures are summarized in Table 2. All PVs were successfully isolated in either group during the index procedure. The vHP-SD ablation mode was exclusively used for all procedures in the vHP-SD group. No switch to conventional mode was necessary to achieve PVI. No differences were observed between the groups with regard to catheter maneuverability and catheter stability along the targeted PVs. CTI block was achieved by vHP-SD only in 4 patients. In three patients (75 %) with a repeat procedure the CTI was checked and was found to be blocked.

**Table 2:** Procedural details: Index procedure.

**Variable**	**vHP-SD**	**Control**	**P**
Number of patients	25	25	
Number of PVs	100	100	
Total number of isolated PVs	100 (1 0 0)	100 (1 0 0)	0.999
First pass isolation rate	21 (84)	22 (88)	0.888
Total procedure time, min	55 (53, 68)	110 (94, 119)	<0.001
Total LA dwelling time, min	42 (36, 44)	83 (64, 110)	<0.001
Total fluoroscopy time, min	6 (5, 9)	12 (9, 16)	<0.001
Total amount of contrast agent, ml	50 (48, 55)	50 (45, 55)	0.562
Total radiofrequency time, sec	328 (277, 392)	1470 (1310, 1742)	<0.001
Total number of applications	80 (69, 98)	81 (56, 108)	0.516
Application duration, sec	4 (4, 4)	22 (14, 25)	<0.0001
Contact force, g	17 (15, 18)	18 (14, 22)	0.613
Power/application, Watt	90 (88, 90)	30 (28, 33)	<0.001
Total delivered power/lesion, Joule	332 (332,336)	598 (462, 703	<0.001
Teso Temp. > 38,5 °C, n	9 (36)	–	–
Max Teso, °C	43 (42, 44)	–	–
Cavotricuspid isthmus block, n	4 (16)	4 (16)	0.999
*Periprocedural complications*			
Severe adverse events	1 (4)	0	0.676
Cardiac tamponade	0	0	0.999
Severe bleeding	1 (4)	0	0.676
Phrenic nerve injury	0	0	0.999
Stroke or TIA	0	0	0.999
Minor complications	2 (8)	1 (4)	0.552
Minor bleeding	2 (8)	1 (4)	0.552
Pericardial effusion	0	0	0.999
Transient air embolism	0	0	0.999
Clinically apparent esophagus injury	0	0	0.999
Charring on catheter tip	0	0	0.999

Values are counts, n (%) or mean (±SD).

PV(s) = Pulmonary vein(s), PVI = pulmonary vein isolation, LA = left atrium, min = minutes, sec = seconds, g = gramm, TIA = transient ischaemic attack.

No differences in terms of serious adverse events or minor complications were observed during the index procedure. Neither documented steam pops, nor catheter tip charring was detected in either of the groups. An esophageal temperature probe was utilized only in the vHP-SD group. A Teso > 38.5 °C was detected in 10 (40 %) patients solely at the posterior part of the left PVs. The median maximum Teso was measured at 43 (42, 44) °C.

### 3.3 Repeat procedures

**Fig. 2:**
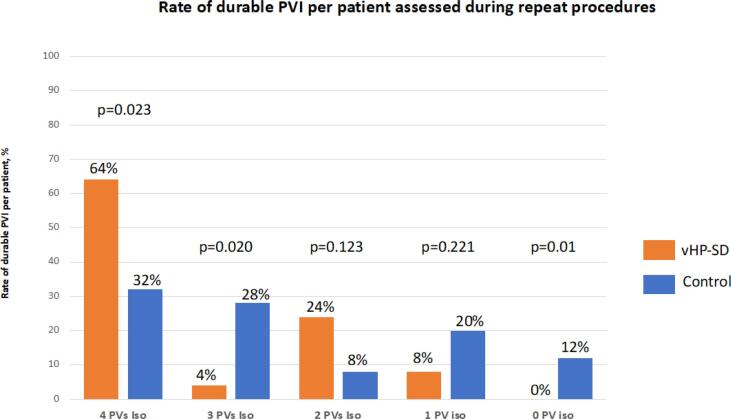
**Findings of repeat procedures (durable PVI per patient)** Comparison of pulmonary vein durability assessed during repeat procedures of the very high-power short-duration group and control group patients.

**Fig. 3:**
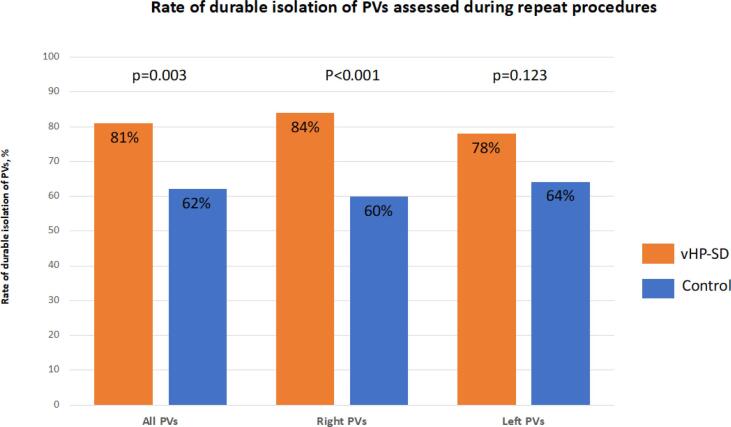
**Findings of repeat procedures (durable isolated PVs)** Comparison of pulmonary vein durability assessed during repeat procedures of the very high-power short-duration group and control group patients.

The findings during repeat procedures are summarized in Table 3**,** Fig. 2 **and** Fig. 3. A total of 25 patients (vHP-SD group) and 25 patients (control group) received a repeat procedure and verification of PVI due to recurrence of AF, AT, typical atrial flutter or LAA closure. The median time to the electrophysiological procedure was 5 (2, 10) months for the vHP-SD group and 8 (3, 14) months for the control group (p = 0.212). Chronic durability of all PVs was assessed in vHP-SD: 16/25 (64 %) and control: 8/25 (32 %) patients (p = 0.023) and vHP-SD: 81 % and control: 62 % of PVs were found to be isolated (p = 0.003). For right PVs vHP-SD: 84 % vs. control: 60 % of PVs (p < 0.001) and for left PVs vHP-SD: 78 % vs. control: 64 % (p = 0.123) were found to be isolated. All PVs were reisolated and ostial potentials were ablated utilizing RF energy. In patients with AT a high-density activation mapping utilizing a 3D electroanatomical mapping system (Carto or EnsiteX) of the LA was assessed and the AT was treated according to the institutional standards.[13] The ablation strategies during repeat procedures are mentioned in Table 3. Briefly, perimitral AT was detected in 6 patients (vHP-SD) and 3 patients (control) and was treated by performing an anterior line due to scar formation at the anterior aspect of the LA. Additionally a mitral isthmus line was deployed in 3 patients (vHP-SD) and 1 patient (control). Due to posterior scar formation a box-lesion was performed in 6 patients (vHP-SD) and 3 patients (control).

**Table 3:** Finding during repeat procedures.

**Variable**	**vHP-SD**	**Control**	**P**
Number of patients	25	25	
*Repeat procedure due to:*			
AF recurrence	10 (40)	16 (64)	0.089
Atrial tachycardia	10 (40)	5 (20)	0.123
Typical atrial flutter	1 (4)	2 (8)	0.552
PV isolation verified during LAA closure	4 (16)	2 (8)	0.384
Time to repeat procedure, months	5 (2, 10)	8 (3, 14)	0.212
PVs	100	100	
Patients with durable complete PVI	16 (64)	8 (32)	0.023
Isolated PVs	81 (81)	62 (62)	0.003
Isolated right PVs	42 (84)	30 (60)	<0.001
Isolated left PVs	39 (78)	32 (64)	0.123
CTI block (index procedure)	4 (16)	3 (12)	0.684
CTI block verified (repeat procedure)	3 (75)	2 (67)	0.809
*Ablation strategy during repeat procedure*			
Reisolation of not isolated PVs	19 (1 0 0)	38 (1 0 0)	0.999
Anterior line	6	2	0.123
Box-lesion	6	3	0.269
Mitralisthmus line	3	1	0.297
LAA isolation	3	1	0.297
CTI block	6	2	0.123

Values are counts, n (%), mean (±SD) or median (interquartile range) as appropriate.

AF = atrial fibrillation, CTI = Cavotricuspid isthmus block, LAA = left atrial appendage, PV(s) = Pulmonary vein(s), PVI = pulmonary vein isolation

In case of previously documented or periprocedural detected typical atrial flutter a CTI block was performed (6 patients (vHP-SD) and 2 patients (control). In patients with CTI block during the index procedure the chronic block was assessed by adequate pacing maneuvers. In patients of the vHP-SD group 3/ 4 (75 %) while in patients of the control group 2/ 3 (67 %) of CTI were found to be durable blocked (p = 0.809).

## 4 Discussion

This study aims to assess chronic PVI durability in patients with previous PVI utilizing solely the vHP-SD mode (QMODE + ) of the QDOT Micro catheter by utilizing a very close protocol.[9] The data was compared to patients with repeat procedures after initial standard AI guided RF ablation. The major findings were:

1) Acute PVI could be achieved utilizing vHP-SD only, with no necessity to switch the ablation mode to moderate power.
2) Total RF time, procedure time and LA dwelling time were significantly reduced utilizing vHP-SD only.
3) Periprocedural complication rate was low with no differences between the groups.
4) During repeat procedures a higher rate of durable PVI was observed for vHP-SD.

### 4.1 Very high-power short-duration catheter ablation

Although the number of single-shot devices for PVI are increasing, the gold standard remains RF based point by point ablation. The advantages for single shot devices are shorter procedure times, learning curves and safety aspects.[14], [2] An improvement of the performance of RF was recently shown for the vHP-SD concept utilizing 90Watts for 4 s in a temperature-controlled mode (QMODE + ) which is realized by the QDOT Micro catheter.[7] The six thermocouples of this catheter enable precise temperature measurement and power modulation to avoid tissue overheating, collateral damage, catheter tip charring and steam pops.

The lesion formation of vHP-SD ablation creates lesions with a lesser depth and larger diameter despite similar total lesion volumes compared to conventional RF lesion formation.[6] Therefore, we recently suggested an adapted, individualized and tighter “very close-protocol” of 3–4 mm ILD at the anterior aspect and 5–6 mm at the posterior aspect of the left atrium using vHP-SD only to achieve safe and fast PVI. PVI utilizing the QMODE + ablation mode provides similar acute success, high first pass isolation rates, short procedure and RF times and low periprocedural complications rates when compared to the standard CF-sensing AI guided PVI.[9], [15], [16], [17].

### 4.2 Chronic PVI durability

With 81 % durable isolated PVs and 64 % of patients showing all 4 PVs durable isolated this rate was unexpectedly high compared to 62 % and 32 % for the control group. Since the rate of durable PVI was higher and a significant difference for durable isolated PVs was only observed for the right PVs as well the observed positive effect of vHP-SD ablation in comparison to control was mainly driven by a better chronic lesion set for the right sided PVs.

Utilizing conventional RF ablation via the close protocol Pooter et al. showed PVI durability of all 4 PVs in 62 % of patients which is similar to our findings.[18] Recently cardiac MRI based late gadolinium enhancement demonstrated homogenous and transmural LA scar with homogeneous and contiguous lesion formation in all PV segments. Complete PV encirclement was observed in 76.7 % for RPVs, in 76.7 % for LPVs, and in 66.7 % for both PV pairs.[10] In this study by Sciacca et al. cardiac MRI was conducted according to the institutional standard 3 months after ablation and was not related to AF recurrence. Although this data is very promising it reflects PVI durability data independently of the status of AF recurrence.[10] Data on PVI durability for the cryoballoon showed 56–69 % durable isolated PVs while all 4 PVs were shown to be isolated in only 21–26 % of patients.[12], [19] The observations on vHP-SD with relatively high rates of durable PVI compared to conventional PV is strengthening the high efficacy of the vHP-SD only strategy utilizing a very close protocol.

Latest findings for chronic durability assessed during repeat procedures after initial pulse field ablation (PFA) based PVI found a rate of 61 % durable isolated PVI,[20] which is much lower in comparison to the initial published rate of 96 %.[21], [22] Although PFA may offer a highly selective ablation modality with safe and fast ablation procedures and promising PVI durability latest findings suggest that more data is necessary to draw final conclusion.

The main concern about vHP-SD is the lack of transmurality in regions with thicker tissue like the CTI. However, Schillaci et al. evaluated vHP-SD ablation of the CTI in 28 consecutive patients and compared the data to patients who underwent CTI ablation by conventional RF ablation guided by AI (control). Here vHP-SD ablation showed similar effectiveness in achieving acute CTI block, with a shorter RF time and similar procedure duration.[23] With 75 % (vHP-SD) and 67 % (control) of proven durable CTI block assessed during repeat procedures our findings support these observations and might be a preliminary hind that vHP-SD ablation might represent an effective and safe strategy to achieve bidirectional CTI block for the treatment of typical AFL.

The higher rate of chronic PVI for the vHP-SD group is potentially related to the following observations: A higher but statistical not significant rate of AF was observed for the control group, while a higher but statistical not significant rate of AT was observed for the vHP-SD group. Additionally, the time to the repeat procedure was shorter but statistical not significant for the vHP-SD group (5 (2, 10) vs. 8 (3, 14) months (p = 0.212). These observations are in line with the observed effect of a higher rate of chronic PVI durability for the vHP-SD group.

With a median procedure time < 60 min the vHP-SD strategy offers short procedures times comparable to single shot devices.[9], [24], [25], [26] With a total median RF time of 328 s this was massively reduced compared to the control group (1470 s). These observations including potentially similar or even faster PVI compared to single-shot-based ablation, the ability to perform further ablation strategies like CTI block and linear ablation applications as well as an excellent safety profile, vHP-SD has the potential for an ideal ablation tool.[9], [13].

### 4.3 Limitations

This study is the first prospective analysis on chronic PVI durability of vHP-SD only based PVI in comparison to standard AI guided PVI assessed by invasive mapping. It is a non-randomized analysis resulting in potential biases. Although we are presenting single-center experience with a relatively small number of patients consecutive patients where prospectively evaluated. Assessment of PVI durability was performed utilizing 3D electroanatomical mapping in 86 and 92 % of patients, however in some cases it was evaluated utilizing only a spiral mapping catheter. This fact is limiting the results since a pure electroanatomical mapping approach might assess more precise data. The number of redo-procedures was relatively low, however we are presenting the first data on PVI durability after vHP-SD based PVI in comparison to conventional RF PVI. No correlation of the individual reconduction gap and contact force, ablation index or stability could be performed due to limited data. This fact is limiting the findings of this study.

## 5 Conclusions

PVI solely utilizing vHP-SD via a very close-protocol provides high rates of chronically isolated pulmonary veins. The data is promising and is comparable to the data of recent single-shot catheter ablation procedures.

## CRediT authorship contribution statement

**Christian-H. Heeger:** . **Behnam Subin:** Data curation, Writing – review & editing. **Charlotte Eitel:** Writing – review & editing. **Sorin Ștefan Popescu:** . **Huong-Lan Phan:** Resources. **Roman Mamaev:** Data curation. **Lorenzo Bartoli:** Data curation. **Niels Große:** Data curation. **Samuel Reincke:** Writing – review & editing. **Anna Traub:** . **Delgado Lopez:** Data curation. **Bettina Kirstein:** Writing – review & editing. **Sascha Hatahet:** Writing – review & editing. **Karl-Heinz Kuck:** Supervision, Writing – review & editing. **Julia Vogler:** Supervision. **Roland R. Tilz:** Supervision.

## Declaration of competing interest

The authors declare that they have no known competing financial interests or personal relationships that could have appeared to influence the work reported in this paper.
